# Supplementary material for: Processive chitinase is Brownian monorail operated by fast catalysis after peeling rail from crystalline chitin
Source: Nat Commun. 2018 Sep 19;9:3814. doi: 10.1038/s41467-018-06362-3 (PMC6145945; doi:10.1038/s41467-018-06362-3)
Supplement: Supplementary file 1 — Supplementary Information [file 41467_2018_6362_MOESM1_ESM.pdf]

Supplementary Information for

Processive chitinase is Brownian monorail operated by fast catalysis after peeling rail from crystalline chitin

Akihiko Nakamura, Kei-ichi Okazaki, Tadaomi Furuta, Minoru Sakurai, Ryota Iino

*Correspondence and request for materials should be addressed to A.N. (aki-naka@ims.ac.jp) or R.I. (iino@ims.ac.jp)

This PDF includes:

Supplementary Figure 1-12

Supplementary Table 1

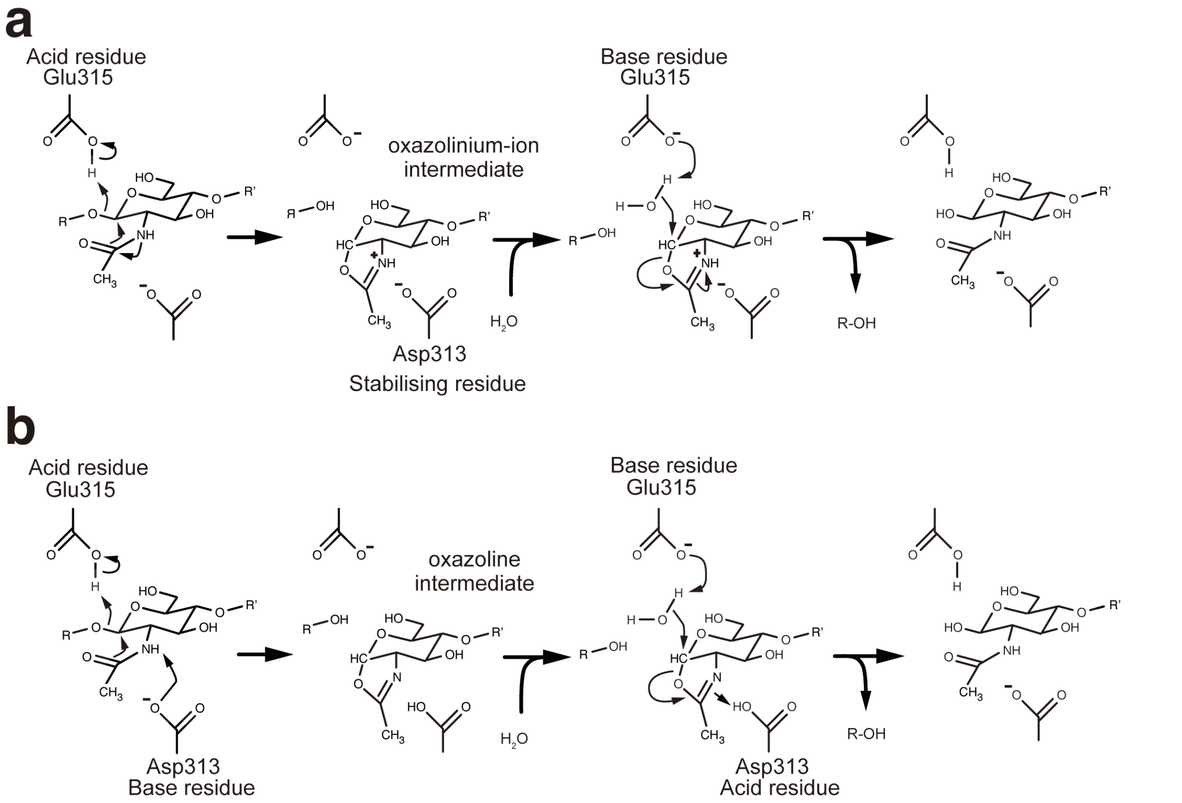

Supplementary Figure 1. Proposed mechanisms of substrate-assisted catalysis of SmChiA. a, Mechanism with oxazolinium-ion intermediate. **b**, Mechanism with oxazoline intermediate.

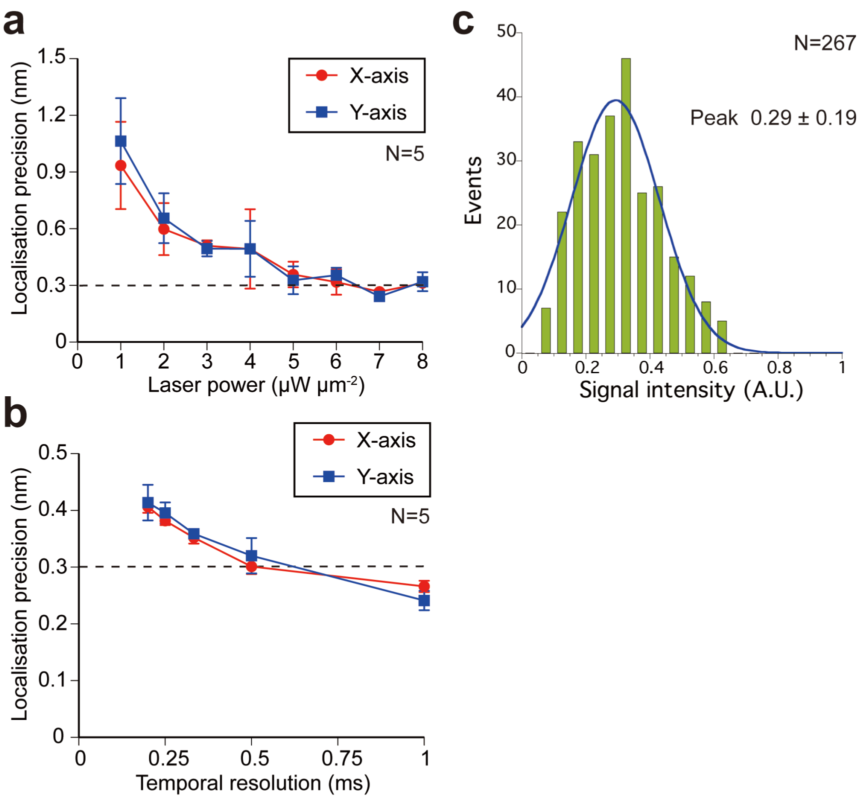

Supplementary Figure 2. Localisation precision of 40-nm AuNP immobilised on glass surface and distribution of signal intensity of 40-nm AuNP. Bare AuNPs were immobilised on a KOH-washed coverslip with 100 mM NaCl and observed with total-internal reflection dark-field microscopy. **a**, Localisation precision at 1.0 ms temporal resolution with various laser powers. Average ± SD of 5 particles were shown. **b**, Localisation precision at various temporal resolution with 7.0 μW μm^{-2} laser power. Localisation precision of the imaging system was 0.3 nm at 0.5 ms temporal resolution. Red circles are X-axis and blue squares are Y-axis. Average ± SD of 5 particles were shown. **c**, Distribution of signal intensities of bare 40-nm AuNPs observed at 0.5 ms temporal resolution with 7.0 μW μm^{-2} laser power. Signal intensities (arbitrary unit, A.U.) were calculated as average of 8 pixels × 8 pixels ROI containing single spot and normalised by the maximum intensity of 12-bit camera (4095 counts). The value is peak ± SD of Gaussian fitting (blue line).

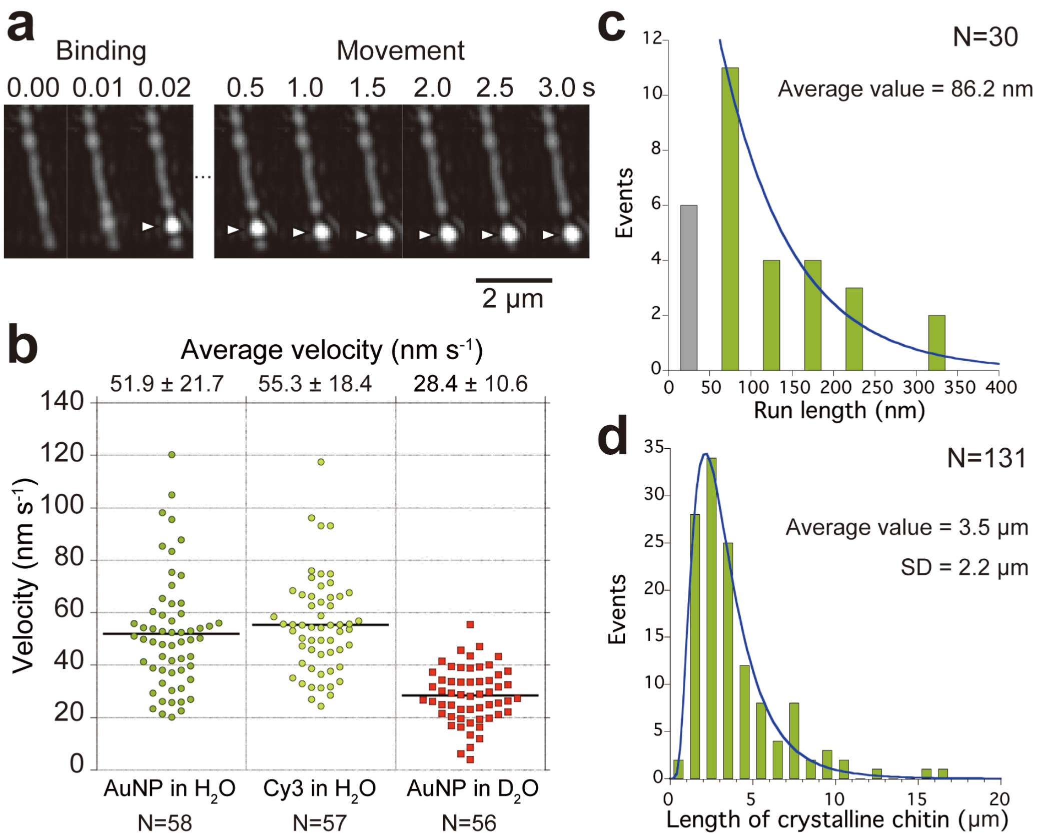

Supplementary Figure 3. Velocity and run length of SmChiA and length of crystalline chitin. a, Example of binding and subsequent movement of SmChiA labelled with 40-nm AuNP on crystalline chitin. After the binding, SmChiA moved downward. **b,** Comparison of velocities of SmChiA labelled with 40-nm AuNP (N=58, dark green circle) or Cy3 (N=57, light green circle), observed in H_2O or D_2O (N=56, red square). The values are mean ± SD. No significant difference was found between the velocities of SmChiA labelled with 40-nm AuNP or Cy3 by two-tailed t-test with a significance level of 5% (t (113) = 0.89). **c**, Distribution of run length of SmChiA labelled with 40-nm AuNP. 1st bin was omitted from the fitting with single exponential decay (blue line), due to uncertainty of short movement. Average value obtained by the fitting is shown. **d**, Distributions of the length of crystalline chitin. Crystalline chitins were observed at 2,000 fps with 7.0 μW μm^{-2} laser power. Average and SD values obtained by the fitting with log-normal distribution function (blue line) are shown.

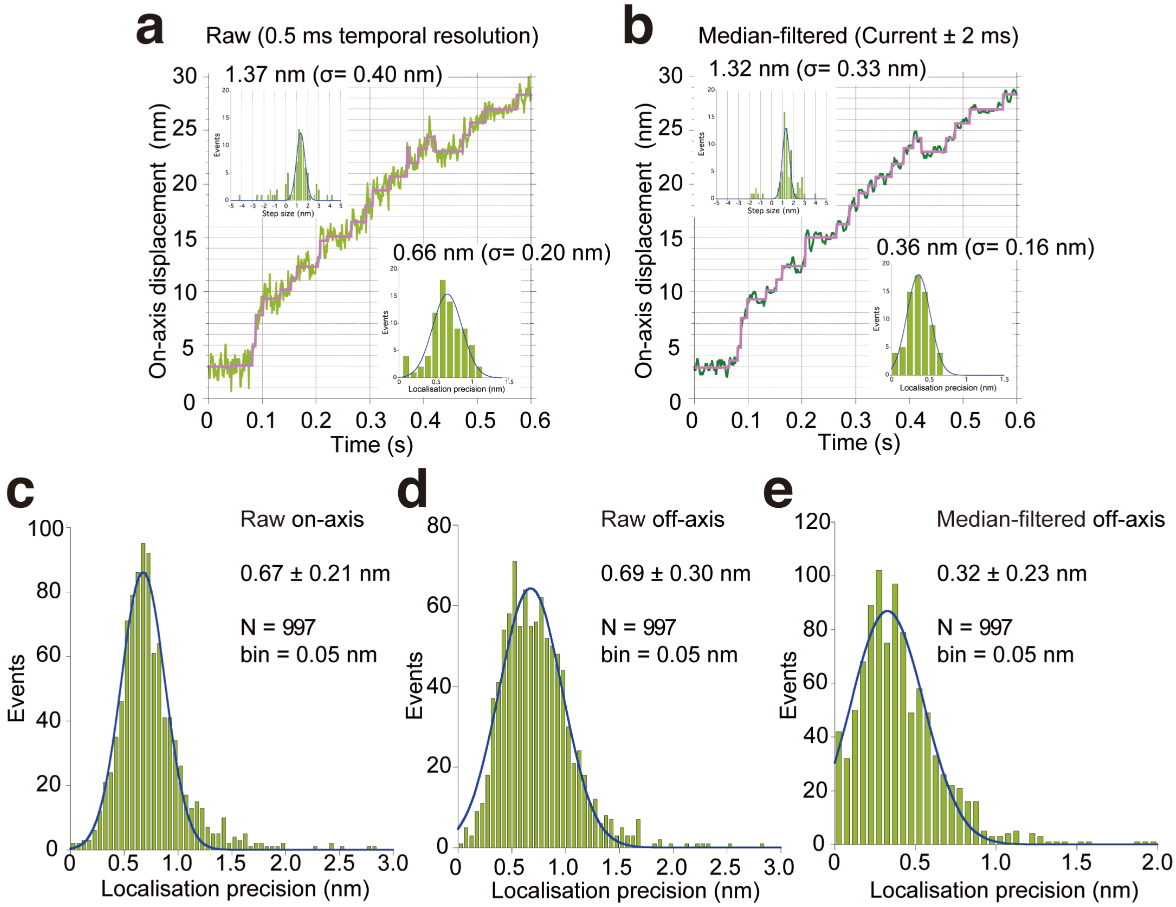

Supplementary Figure 4. Effect of median filtering of raw trace on step detection and localisation precision. a and **b,** Green lines are raw (**a**) or median-filtered (current ± 2 ms, **b**) traces and magenta lines are steps detected by the step-finding algorithm. Top left insets show step size distributions and bottom right insets show distributions of localisation precision during detected pauses. **c** and **d**, Distributions of localisation precision during pauses of moving molecules, calculated from raw trajectories in the on-axis (**c**) and off-axis (**d**). **e**, Distributions of localisation precision during pauses of moving molecules, calculated from median-filtered trajectories in the off-axis. That of on-axis is shown in Fig. 2e. The values in **c** to **e** are peak ± SD of Gaussian fittings (blue lines).

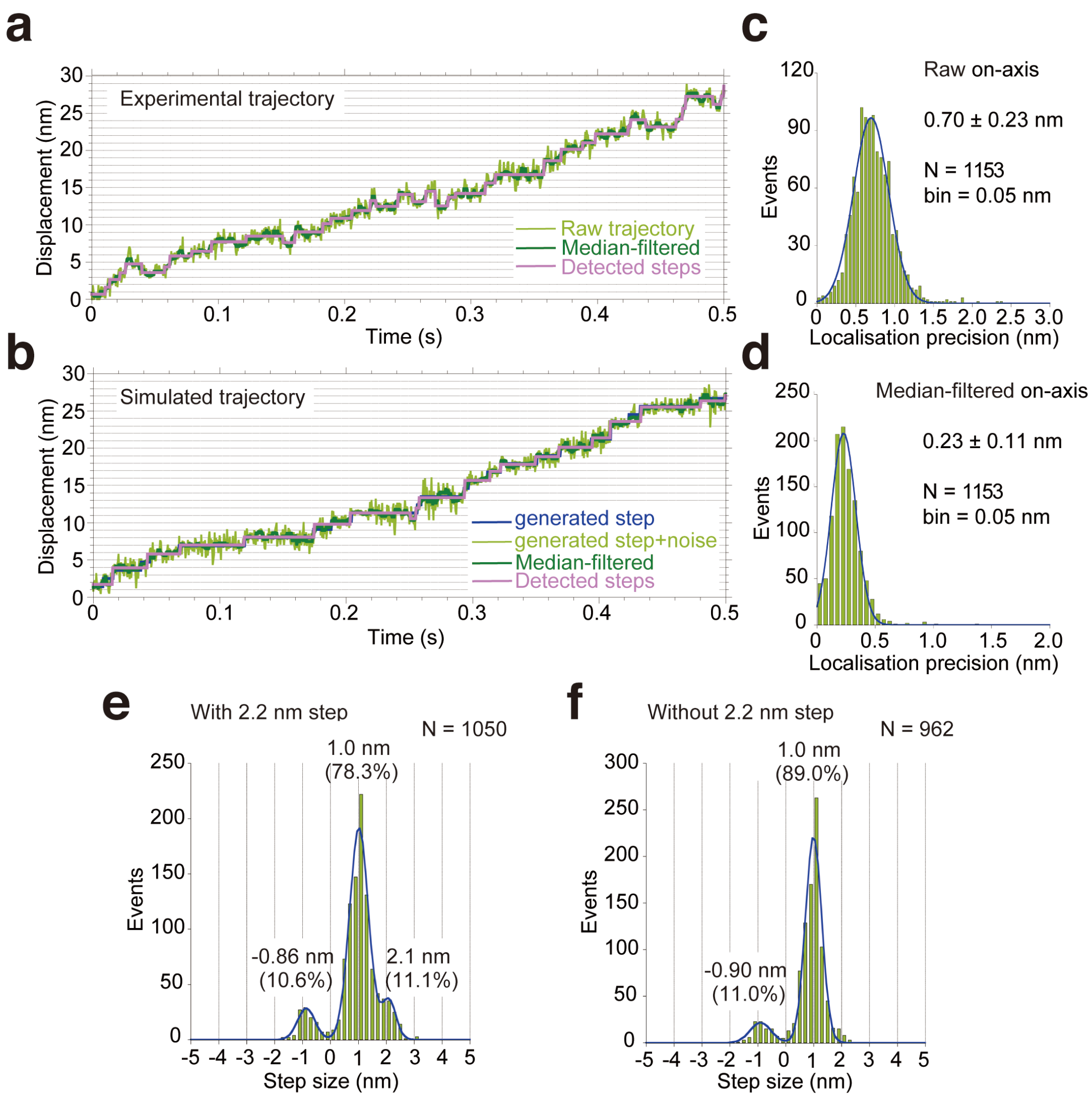

Supplementary Figure 5. Analysis of a simulated trajectory. a and **b**, Examples of experimental and simulated trajectory. Simulated trajectory is generated using the localisation precision, step sizes and ratio, and kinetic parameters obtained by the experiments. Blue line is generated steps from parameters. Light green lines are experimental-raw trajectory or simulated steps with noise. Dark green lines are median-filtered traces, and magenta lines are steps detected by the step-finding algorithm. **c** and **d**, Distributions of localisation precision during pauses of raw (**c**) and median-filtered (**d**) simulated trajectories detected by the step-finding algorithm. The values are peak ± SD of Gaussian fittings (blue lines). **e** and **f**, Distributions of step sizes detected by step-finding algorithm on the simulated trajectories with (**e**) and without (**f**) 2.2 nm steps.

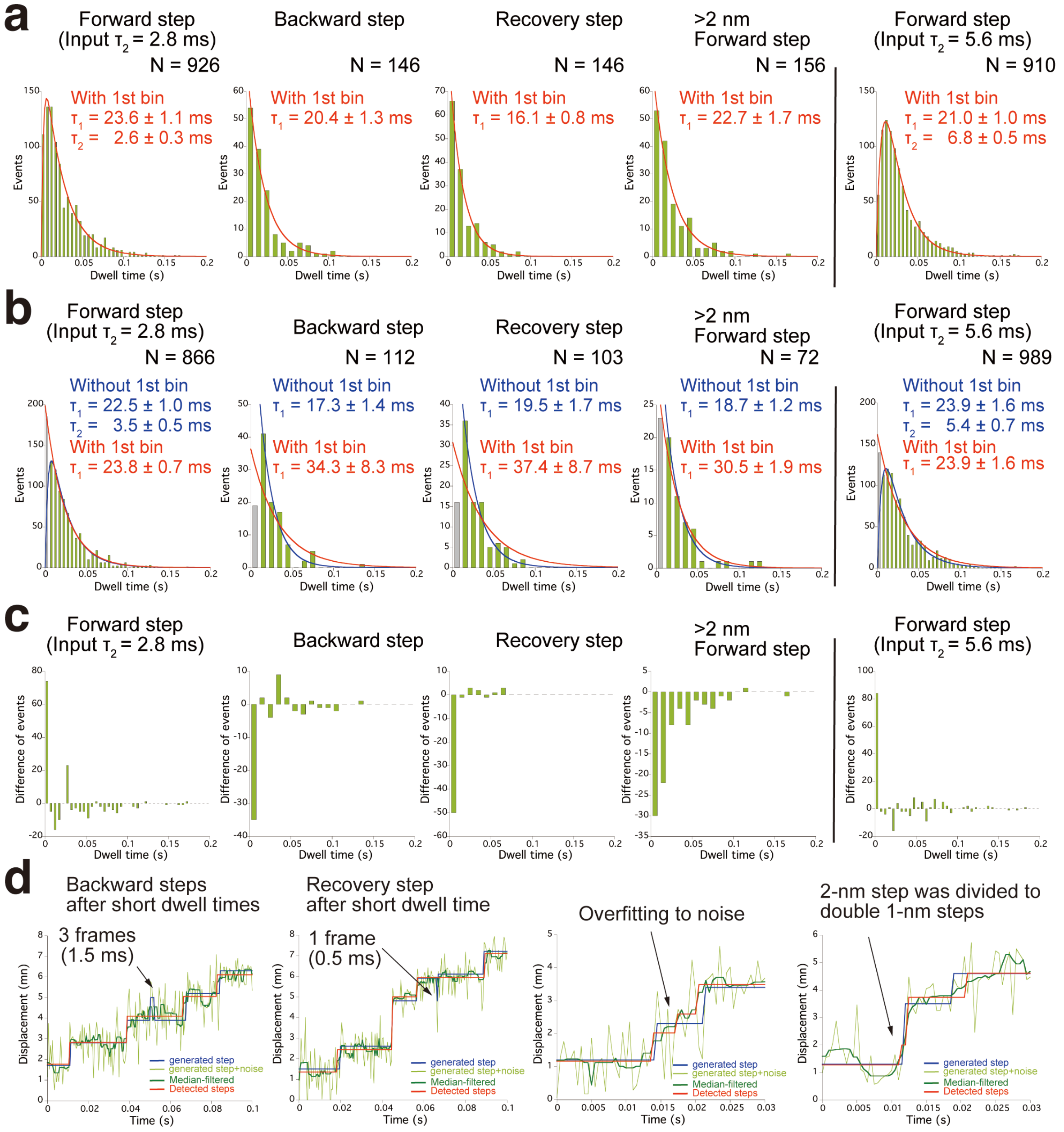

Supplementary Figure 6. Effects of over- and under-fitting by the step-finding algorithm on the dwell time analysis. a and b, Distributions of dwell times before steps generated by simulation without noise (**a**) and detected by the step-finding algorithm applied to simulation with noise (**b**). The values are obtained parameters ± fitting errors. Fittings without and with 1st bin are shown as blue and red lines, respectively, on the

distributions of dwell times. **c**, Differences in number of events (the results of generated steps subtracted from those of the step-finding algorithm) clearly showed that over-fitting of the forward step and under-fitting of backward and recovery steps largely affect the heights of 1st bin. Even in the simulation with artificially lengthened T_2 (= 5.6 ms), the 1st bin height was much larger than that of generated steps, indicating that higher 1st bin of the forward step is due to overfitting, not to insufficient temporal resolution. **d**, Examples of under-fittings to backward and recovery steps (leftmost and 2nd from left) and over-fittings to the noise (2nd from right) and a 2-nm step (rightmost) by step-finding algorithm. Light or dark green lines are raw or median-filtered traces and magenta lines are steps detected by the step-finding algorithm.

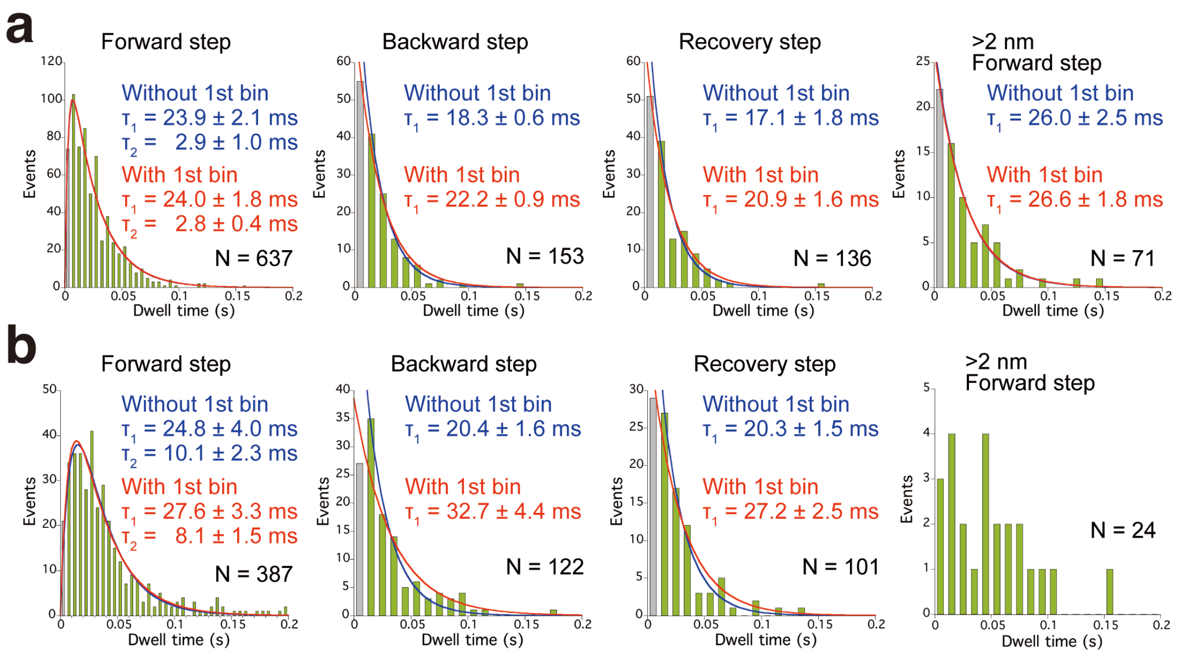

Supplementary Figure 7. Summary of time constants of the dwell time before steps estimated by fittings without and with 1st bins. Dwell times in H_2O (**a**) and those in D_2O (**b**). Fittings without and with 1st bins are shown as blue and red lines, respectively. The values are obtained parameters ± fitting errors.

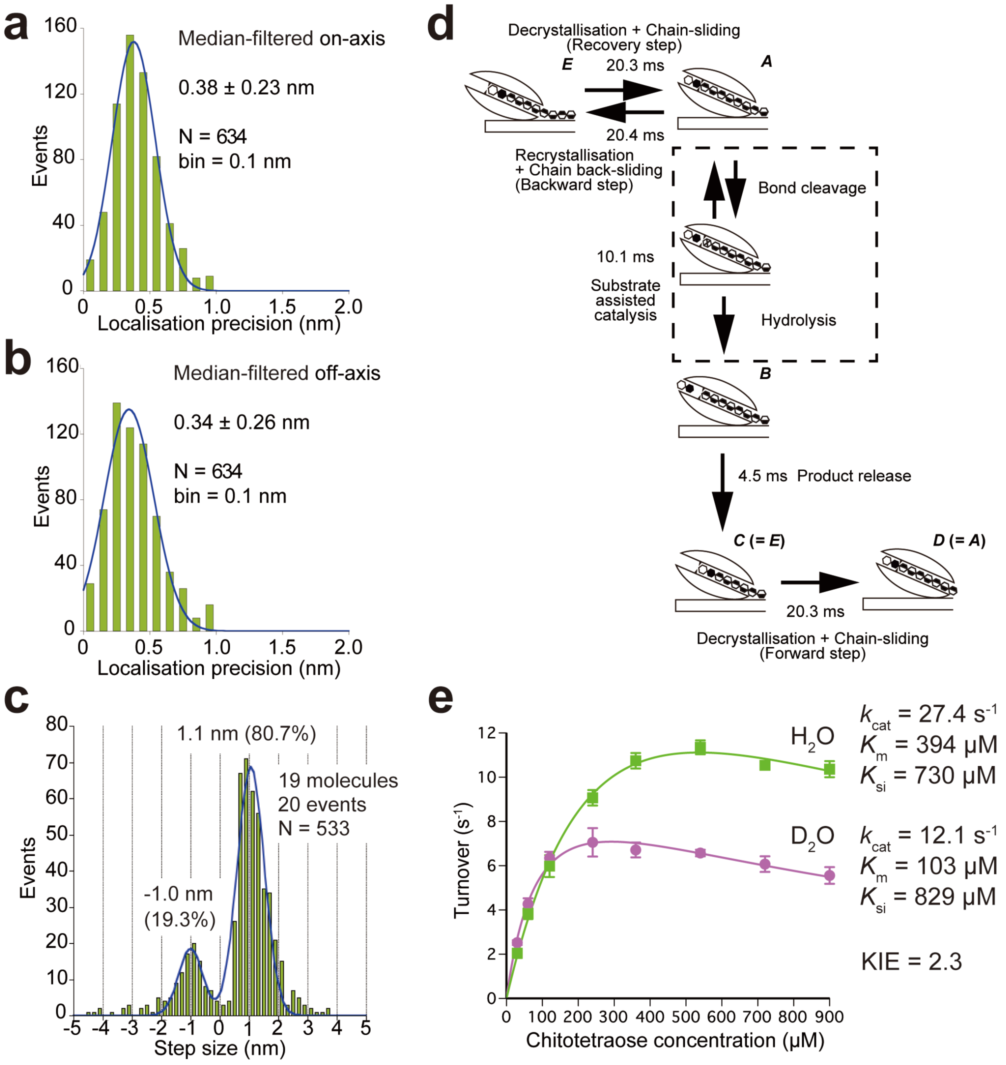

Supplementary Figure 8. Summary of distributions of localisation precision during detected pauses, step sizes and time constants in D_2O. a and **b**, Distributions of localisation precisions after ±2-ms median-filtering along with on-axis (**a**) and off-axis (**b**). The values are peak ± SD of Gaussian fittings (blue lines). **c**, Distribution of step sizes. **d**, Summary of time constants in D_2O. **e**, Plot of chitotetraose hydrolysis activity (turnover) against chitotetraose concentration in H_2O (green squares) or D_2O (magenta circles) with SD of individual triplicate measurements. Plots were fitted by the Michaelis-Menten equation with the substrate inhibition. The kinetic isotope effect (KIE) on the hydrolysis of water-soluble chitotetraose was 2.3.

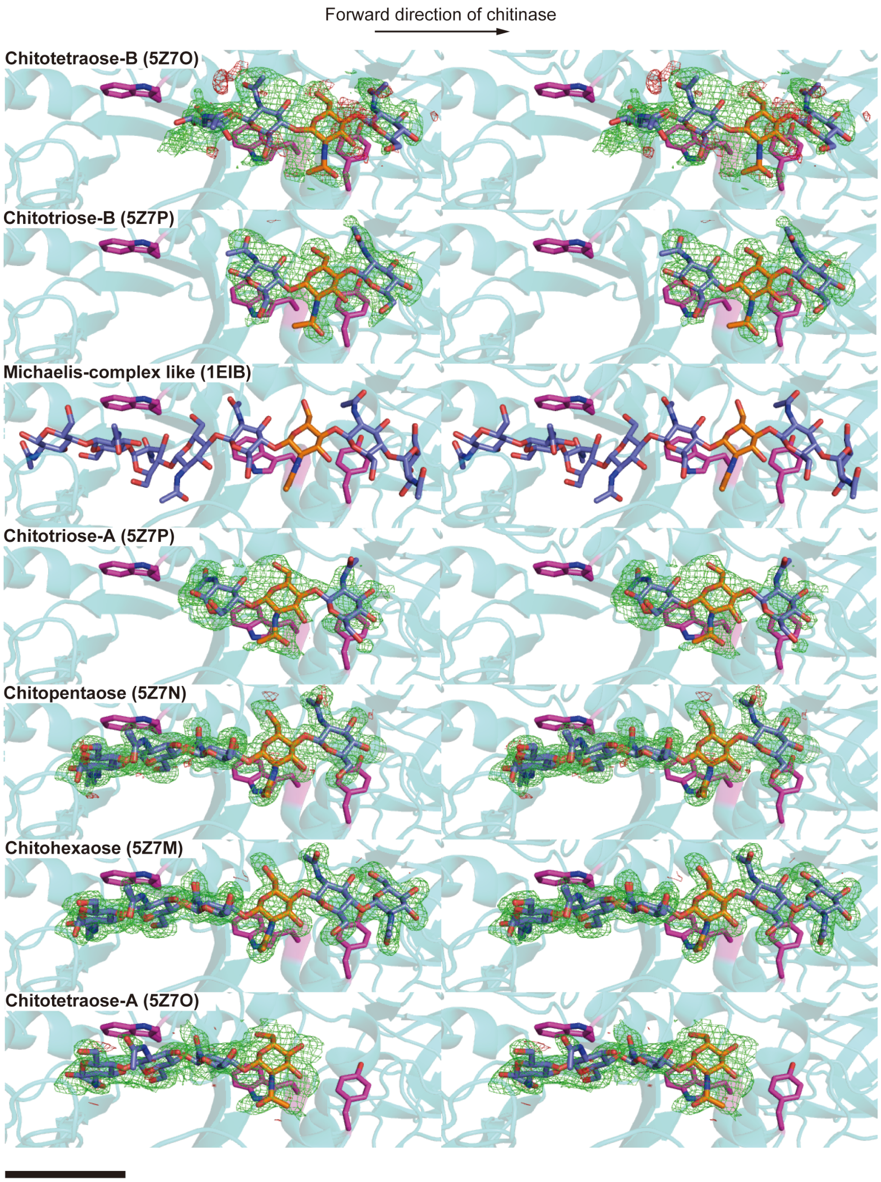

Supplementary Figure 9. Crystal structures of SmChiA with various bound oligosaccharide lengths. Cross-eyed stereo views of the omit maps of the crystal structures calculated without oligosaccharides. The $F_{obs} - F_{calc}$ maps of chito-oligosaccharide molecules are shown at 3 sigma in green and red for positive and negative, respectively. Chitotriose and chitotetraose complexes showed two binding conformers. Scale bar is 1 nm.

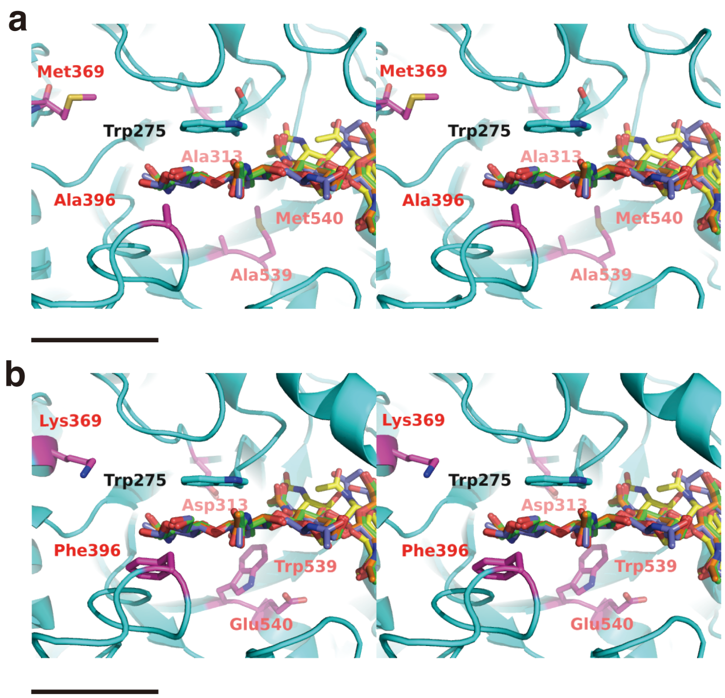

Supplementary Figure 10. Superimposed structure of sliding-intermediate substrates with wild-type enzyme. **a**, Cross-eyed stereo view of the sliding-intermediate structure around mutated amino acid residues. Substituted Ala or Met (D313A, K369M, F396A, W539A, and E540M), shown by stick and purple, did not interact with chito-oligosaccharides. Carbon atoms of chitohexaose and chitopentaose are shown by orange and green, those of chitotetraose and chitotriose are shown by blue and yellow, respectively. Scale bar is 1 nm. **b**, Cross-eyed stereo view of superimposed structure of the sliding-intermediate chito-oligosaccharides with wild-type SmChiA (PDB ID: 1EDQ). Corresponding amino acid residues (D313, K369, F396, W539, and E540) are shown by stick and purple. No steric hindrances between these amino acid residues and chito-oligosaccharides were observed. Carbon atoms of chitohexaose and chitopentaose are shown by orange and green, those of chitotetraose and chitotriose are shown by blue and yellow, respectively. Scale bar is 1 nm.

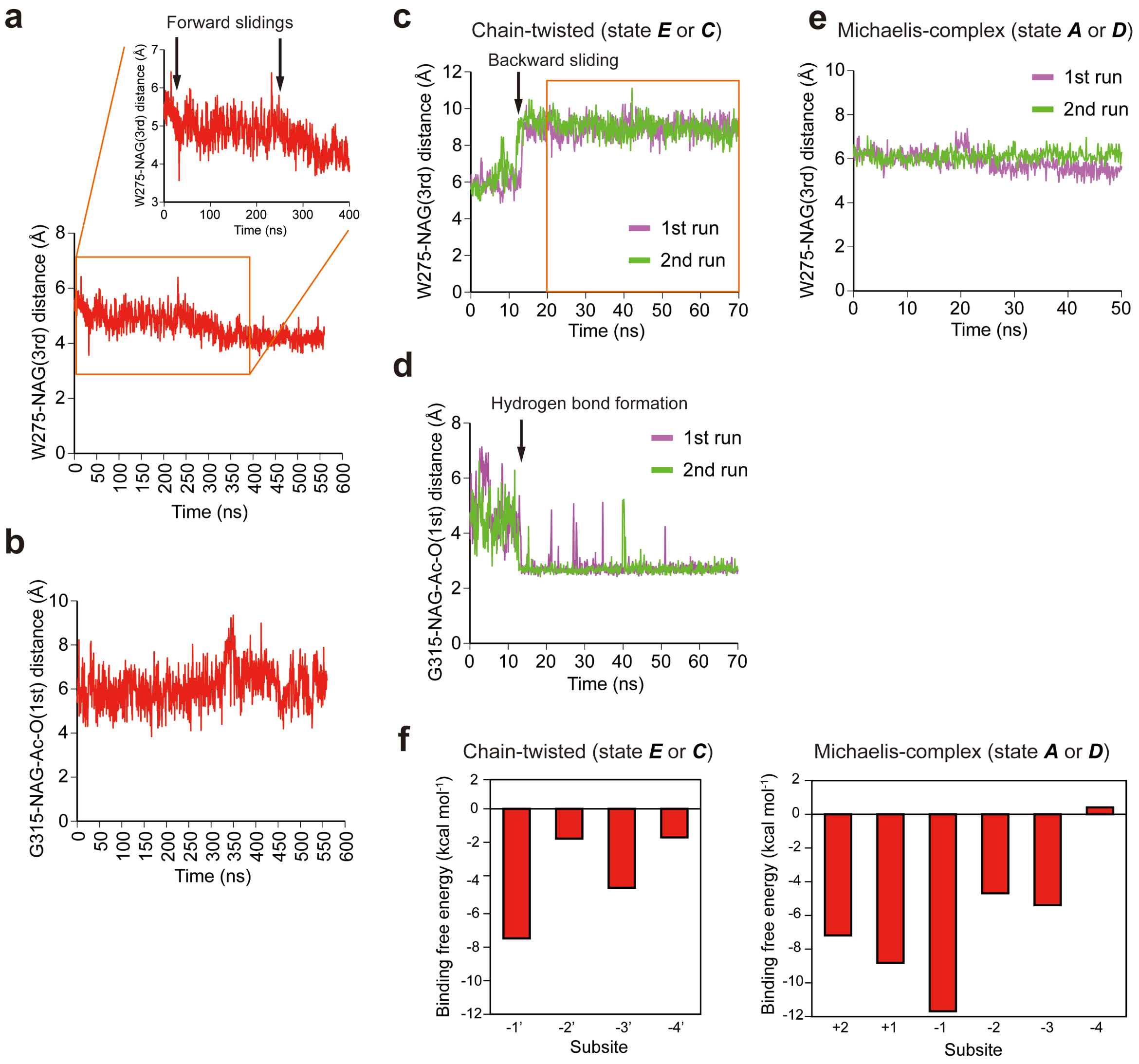

Supplementary Figure 11. Distance trajectories and estimation of binding free energy of the substrate by molecular dynamics (MD) simulation. a, Time course of distance between the centroid of Trp275 side chain and oxygen atom of glycoside bond connecting 2nd and 3rd N-acetyl glucosamines from reducing end in the MD simulations. **b**, Time course of distance between deprotonated oxygen atom of Glu315 side chain (OE1) and oxygen atom of acetyl group at reducing end of chitohexaose during forward sliding of chain. **c**, Time courses of distances the carbon atom (CE2) of Trp275 side chain and oxygen atom of glycoside bond connecting 2nd and 3rd N-acetyl glucosamines from reducing end.

The distances in the Chain-twisted system were converged to ca. 9 Å after 13 ns. Therefore, the last 50-ns periods (orange square) were used for analysis of the binding free energy. **d**, Time courses of distance between protonated oxygen atom of Glu315 side chain (OE1) and oxygen atom of acetyl group at reducing end of chitohexaose during backward sliding of chain. **e,** Time courses of distances the carbon atom (CE2) of Trp275 side chain and oxygen atom of glycoside bond connecting 2nd and 3rd N-acetyl glucosamines from reducing end. In the Michaelis-complex system, all the periods were used for analysis of the binding free energy. Trajectories of 1st and 2nd trials were shown by magenta and green respectively. **f**, Average binding free energies of 2-runs at each subsite, estimated by the MM/GBSA method.

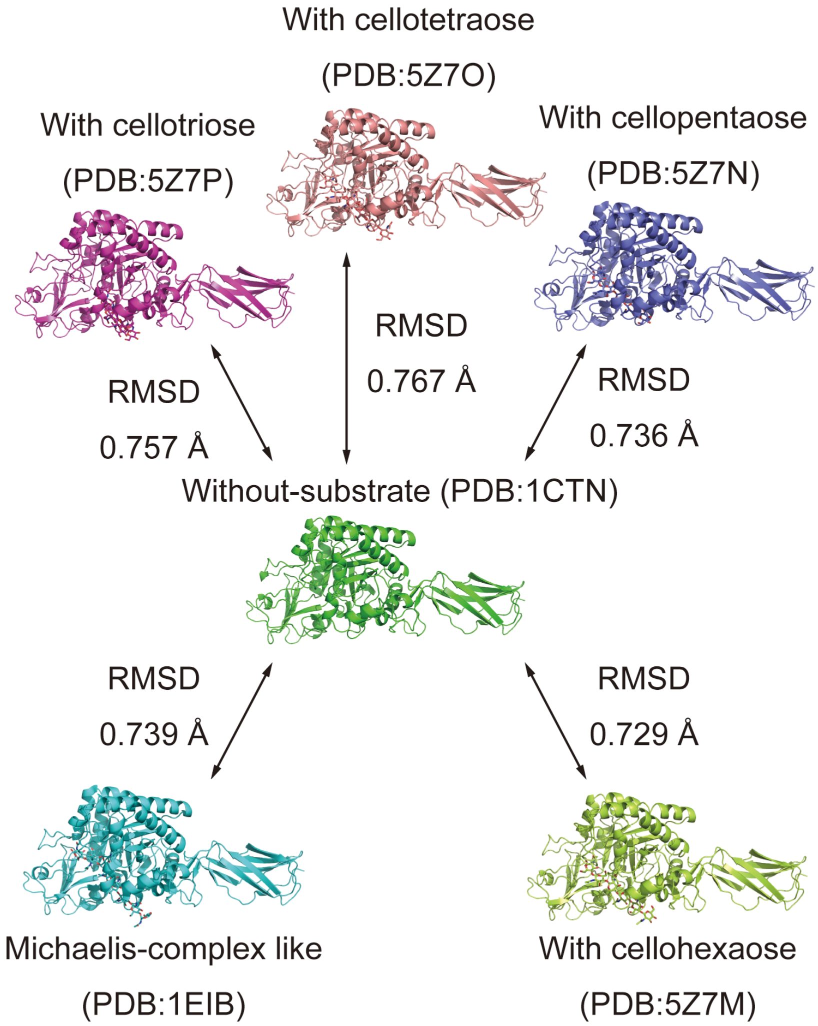

Supplementary Figure 12. Comparison of the crystal structures of SmChiA without and with bound substrates. RMSD between the substrate-free structure (PDB: 1CTN) and the structures with various bound oligosaccharide lengths (PDB: 5Z7M, 5Z7N, 5Z7O, 5Z7P), and the Michaelis-complex-like structure (PDB: 1EIB).

Supplementary Table 1. Primers used for generation of SmChiA mutants

Name	Sequence
ChiA-FaXa_pET27b-F	5'-ATATACATATGCGCAAATTTAATAAACCGCTGTTG-3'
ChiA-FaXa_pET27b-R	5'-CGAGTGCGGCCGCTTAATGATGATGATGATGATGACCACCAAAACGACCT TCGATTTGAACGCCGGCGCTGTTGC-3'
ChiA-plasmid-F	5'-ATCGACGCGGATAACGGCGATATTCTCAACAGCATGAAC-3'
ChiA-plasmid-R	5'-GATATCCACGCCGTCGAAGAACTTCCAGGTCTGCAGGAACTC-3'
ChiA-D313A-F	5'-GACGGCGTGGATATCGCGTGGGAGTTCCCGGGCGGCAAAGGC-3'
ChiA-W539A-E540M-R	5'-GTTATCCGCGTCGATCATCGCGGAGAACAGGCCGCCCAGCTGCTTATCCA ACACGTAC-3'
ChiA-F396A-F	5'-CGCAGAACTCGATGGATCACATCTTCCTGATGAGCTAC+GACTTCTATGGC GCCGCGGATCTGAAGAACCTGGGGCATCAG-3'
ChiA-K369M-R	5'-CAGGAAGATGTGATCCATCGAGTTCTGCGCAACGTTGTAAGCCACCTTGT CGATCATGTCCTTACCGGCGCTGATGGCGG-3'
ChiA-W167A_F	5'-CGTCGAGGCGGGCGTTTACGGGCGCAATTTCACCGTCGACAAG-3'
ChiA-W167A_R	5'-ACGCCCGCCTCGACGAAATAAGAACCGACCACTTTGCCGGAGTTCTGTTT ATACG-3'
