# Supplementary material for: Processive chitinase is Brownian monorail operated by fast catalysis after peeling rail from crystalline chitin
Source: Nat Commun. 2018 Sep 19;9:3814. doi: 10.1038/s41467-018-06362-3 (PMC6145945; doi:10.1038/s41467-018-06362-3)
Supplement: Supplementary file 3 — Description of Additional Supplementary Files [file 41467_2018_6362_MOESM3_ESM.pdf]

Description of Additional Supplementary Files

File Name: Supplementary Movie 1
Description: An example of SmChiA movement probed by 40-nm AuNP observed at 0.5 ms temporal resolution. The image sequence and trajectory are shown in Fig. 2a and b respectively. Movie was prepared by the extracted frames (one per 20 frames), and play speed is 100 fps.

File Name: Supplementary Movie 2
Description: Visualised trajectory of forward chain-sliding from the Sliding-intermediate structure in MD simulation with deprotonated Glu315 at 300K (0 ns to 560 ns in simulation was extracted). Trp275, Glu315 and Phe396 are shown by stick. Play speed is 30 ns trajectory/s.

File Name: Supplementary Movie 3
Description: Visualised trajectory of backward chain-sliding from the Sliding-intermediate structure in MD simulation with deprotonated Glu315 for 150 ns at 300K. Trp275, Glu315 and Phe396 are shown by stick. Play speed is 30 ns trajectory/s.

File Name: Supplementary Movie 4
Description: Visualised trajectory of 1st-run in MD simulation of the Sliding-intermediate to form the Chain-twisted state with protonated Glu315 for 70 ns at 300K. Trp275, Glu315 and Phe396 are shown by stick. Play speed is 10 ns trajectory/s.

File Name: Supplementary Movie 5
Description: Visualised trajectory of 1st-run in MD simulation of the Michaelis-complex for 50 ns at 300K. Trp275 is shown by stick. Play speed is 10 ns trajectory/s.

File Name: Supplementary Movie 6
Description: Summary of chemo-mechanical coupling and operation mechanisms of SmChiA movement on crystalline chitin. Substrate-assisted catalysis with oxazoline intermediate was used in the movie, but the intermediate structure is still open for discussion.
